# 

**Published:** 1894-06

**Authors:** 


					CONTENTS. ut(fi 1897 ,
//?
Somnambulism.
By J. Michell
Clarke, M.A., M.D. Cantab.,
M.R.C.P
81
Adherent Pericardium in Children.
By Theodore Fisher, M.D. Lond.
93
A Case of Syringomyelia.
By F. H.
Edgeworth, M.B., B.A. Cantab.,
B.Sc. Lond
100
Pigmentation in Amenorrhoea.
By
A. E. Aust Lawrence, M.D. Aberd.
107
Progress of the Medina} ^Qiencesip0?,\>'
Medicine.
By i n glrxo y
Smith. M.D.
108
Surgery.
By C. A. Morton
lli
Obstetrics.
By L. M. Griffiths
119
Reviews of Books
122
Notes on Preparations for the Sick
143
Meetings of Societies
149
The Library of the Bristol Medico-
Chirurgical Society  152
The William F. Jenks Memorial Prize
154
The International Congress of
Hygiene and Demography
194
I Scraps
155

				

